# Predicting temperature curve based on fast *k*NN local linear estimation of the conditional distribution function

**DOI:** 10.7717/peerj.11719

**Published:** 2021-07-09

**Authors:** Ibrahim M. Almanjahie, Zoulikha Kaid, Ali Laksaci, Mustapha Rachdi

**Affiliations:** 1 Department of Mathematics, College of Science, King Khalid University, Abha, South Region, Saudi Arabia; 2 Statistical Research and Studies Support Unit, King Khalid University, Abha, South Region, Saudi Arabia; 3 AGIM Team, Laboratoire AGEIS, Universite’ Grenoble Alpes (France), Universit’e Grenoble Alpes, Grenoble, France

**Keywords:** Functional time series, Meteorological data, Local linear fitting, Distribution function, Kernel weighting, Conditional predictive region, k-nearest neighbors smoothing

## Abstract

Predicting the yearly curve of the temperature, based on meteorological data, is essential for understanding the impact of climate change on humans and the environment. The standard statistical models based on the big data discretization in the finite grid suffer from certain drawbacks such as dimensionality when the size of the data is large. We consider, in this paper, the predictive region problem in functional time series analysis. We study the prediction by the shortest conditional modal interval constructed by the local linear estimation of the cumulative function of $Y$ given functional input variable $X$. More precisely, we combine the $k$-Nearest Neighbors procedure to the local linear algorithm to construct two estimators of the conditional distribution function. The main purpose of this paper is to compare, by a simulation study, the efficiency of the two estimators concerning the level of dependence. The feasibility of these estimators in the functional times series prediction is examined at the end of this paper. More precisely, we compare the shortest conditional modal interval predictive regions of both estimators using real meteorological data.

## Introduction

Over the past decades, the volume and complexity of collected data have rapidly increased the sizes and number of covariates. This increase has created significant potential and demand for scientific and technological innovations. The increased storage capacity of information, the improvement of computers and their processing capabilities, the proliferation of surveillance systems, and the improved sensors are the technological progress that has favored the emergence of this kind of data. These are now commonly used in many fields of application such as astronomy, biology, climatology, ecology, chemistry, economics, medicine, engineering sciences, etc. The standard statistical models based on the discretization of the big data in the finite grid suffer from certain drawbacks. The first anomaly is the problem of the dimensionality curse when the size of the discretization grid is large. The second anomaly is that, with the transformation, the original data loses its characteristics such as the functionality, correlation, heteroscedasticity or the homoscedasticity of the data. In particular, the asymptotic behavior of the constructed data is related to the obtained sampling.

Recall that all the mentioned defects can substantially affect the multivariate approaches’ efficiency in big data analysis. To overcome these problems, a new approach in modern statistics called functional data analysis is developed recently. Such procedure allows the use of the natural space of the data, which permits the profit from the whole information. In this modern statistics branch, the local linearity method (LLM) estimation is widely studied. It is motivated by its small bias in the estimation processing (see Fan & Gijbels (1996) for a uni-dimensional framework, and Baìllo & Grané (2009), for the Nonparametric Functional Data Analysis (NPFDM) set up in the area of functional statistics). Barrientos-Marin, Ferraty & Vieu (2010) studied the LLM-estimation of the nonparametric operator of the Banach explanatory variable. We cite Berlinet, Elamine & Mas (2011) for an alternative LLM-estimator constructed by inverting the local variance-covariance matrix of the functional variable. Concerning the LLM-estimation of the conditional cumulative distribution function (CCDF), we point out that the first result was stated by Laksaci, Rachdi & Rahmani (2013). Later, Demongeot et al. (2014) precise the least square error of the LLM-estimator of the CCDF-model. We also point out that the previous studies utilized the kernel local linearity method. However, this paper focuses on CCDF-estimation with a new weighting approach obtained by mixing the local linear fitting to the *k*-Nearest Neighbors (*k*NN) method. Indeed, the method of *k*NN has been received growing attention in nonparametric functional statistics. In particular, the first results on the kNN-LLM estimation were obtained by Chikr-Elmezouar et al. (2019). They studied the conditional density estimation using the local linear method under the kNN smoothing. They established the almost complete consistency of the obtained estimator. Recently, Almanjahie et al. (2021) consider the estimation of the robust regression function using the kNN. They proved the uniform consistency on the number of the neighbor of the constructed estimator. We also mention Laksaci, Ould-Said & Rachdi (2021) for the kNN estimation of the quantile regression. For more recent results on the kNN-LLM estimation in functional statistics, we cite Rachdi et al. (2021). They treated the case when the response variable is observed with missing at random and the regressor is of functional nature.

On the other hand, the estimation based on the *k*NN method has more advantages than the Nadaraya–Watson algorithm (see Burba, Ferraty & Vieu (2009), for more discussion on the motivations on this approach). In this paper, we benefit from the advantages of both the kNN weighting and LLM-fitting by combining the two algorithms to provide a fast efficiency estimator for the CCDF. Specifically, we combine these approaches to construct two estimates of the CCDF and to the shortest conditional modal interval (SCMI) predictive regions using mixing functional time series. Notice also that the smoothing parameter in the *k*-Nearest Neighbors method is varied randomly with respect to the observations. This feature makes the applicability of these estimators very fast and accurate because the smoothing parameter is selected with respect to the nature of the data. To highlight the smoothing parameter selection issue, we compare several cross-validation rules to select the best number of the neighborhood. The superiority of both estimators in practice is emphasized by the meteorological data; specifically, the constructed estimator to predict the yearly curve of the temperature in Europe central.

This paper is structured as follows. The two kNN-LLM estimators of the CCDF are constructed in the “Method” section. We devote the section “Results and discussions” to some discussions related to our proposed estimators. The simulation study for testing the superiority of the proposed estimators is presented in the “Simulation study” section. The performance of the constructed estimator in temperature prediction using real data is conducted in the “Real-data application” section. Our conclusion and remarks for further research are presented in the last section.

## Methods

In this section, we will construct two new estimators by combining the local linear approach to the kNN smoothing methods.

### The fast kNN-LLM of the CCDF

Consider (*X*_1_,*Y*_1_), (*X*_2_,*Y*_2_), (*X*_3_,*Y*_3_)…, (*X*_*n*_,*Y*_*n*_) be stationary sequence of random vector (*X*, *Y*) valued in ${\rm {\cal F}} \times {\rm I}{\rm R}$, where ${\rm {\cal F}}$ is a separable metric and has a metric *d*. Let *N*_*x*_ be the neighborhood of fixed curve $x \in {\rm {\cal F}}$, for which we suppose that the conditional cumulative distribution function (CCDF) *F*(·|*x*) has a continuous conditional density *f*(·|*x*).

This estimation procedure is based on the definition of CCDF, as conditional expectation:

$F(y|x)={ \mathrm {I\!E}}\left[\hbox{ 1\hskip -.19pcI} _{\{Y_i\leq y\}}\big|X=x\right]$

with $\hbox{1\hskip -.19pcI}$_*A*_ is the indicator function of *A*. In the LLM technique, we approximate *F*(*y*|*x*) locally in *N*_*x*_ using

(1) $\forall {x_0} \in {N_x},\quad F(y|{x_0}) = {a_{yx}} + {b_{yx}}d({x_0},x) + o(d(x,{x_0})).$

So, the *k*NN-LLM estimator of CCDF *F*(*y*|*x*) is obtained by estimating *a*_*yx*_ and *b*_*yx*_, in (1), as the minimizers of the rule,

$Min_{a,b}\sum_{i=1}^n \left(\hbox{1\hskip -.19pcI} _{\{Y_i\leq y\}}-a-bd(X_i,x)\right)^{\! 2}Ker\left(\frac{d(x,X_i)}{  {I\!H}_k}\right),$

where *Ker* means the kernel function and ${ \mathrm {\rm I\!H}} = \min \{ h \in{ \mathrm {\rm I\!R}} ^ + ,\;\text{satisfies}\; {\sum\nolimits_{i = 1}1\hskip -.19pcI _{Ba(x,h)}}({X_i}) = k\}$. Similarly to Barrientos-Marin, Ferraty, & Vieu (2010), we obtain by derivative that the $\widehat a$ and $\widehat b$ are the solutions of

$^tL(Ker\Upsilon-Ker\, L)\left(\matrix{\widehat{a}\\\widehat{b}\\}\right)=0,$

where

$^tL=\left(\matrix{1,1,\ldots,1\\cr d(X_1,x)\ldots,d(X_n,x)\\}\right)$

and

$KER=diag\left( Ker\left(\frac{d(x,X_1)}{ \mathrm {I\!H}_k}\right), Ker\left(\frac{d(x,X_2)}{ \mathrm {I\!H}_k}\right), \ldots Ker\left(\frac{d(x,X_n)}{ \mathrm {I\!H}_k}\right) \right) \quad$

with

$\mbox{and} \quad ^t\Upsilon=\left({\rm 1\hskip -.19pc I} _{\{Y_1\leq y\}},\ldots, {\rm 1\hskip -.19pc I}_{\{Y_n\leq y\}}\right).$

It follows that

$\left(\matrix{\widehat{a}\\\widehat{b}\\}\right)=(^tLKER\, L)^{-1}(^tL\,KER\Upsilon).$

Hence,

$\widehat{a}=(1,0) (^tLKerL)^{-1}(^tLKer\Upsilon).$

Finally, the Fast kNN-LLM of the CCDF *F*(*y*|*x*) is

$\widehat{F} (y|x)= \widehat{a} _{yx}= \frac{\sum_{{i,j=1}}^n \beta_{ij} {1}_{\{Y_1\leq y\}}\over{\sum_{{i,j=1}}^n \beta_{ij}}},$

where

$\beta_{ij}=d(X_i,x)\left(d(X_i,x)-d(X_j,x)\right)\times Ker({ \mathrm {\rm I\!H}}_k^{-1}d({ \mathrm {\rm I\!H}}X_i))Ker({ \mathrm {\rm I\!H}}_k^{-1}d(x,X_j)).$

### The smooth *k*NN-LLM of the CCDF

An alternative estimation of CCDF is built by treating the function *F*(·|*x*) as a conditional expectation, i.e.,

${\mathrm {I\!E}}[H(\ell^{-1}(y-Y_i))\big|X_i=x]\to F(y|x)\hbox{ as }\ell\to 0,$

where *H* is the cumulative distribution function, $(\ell_n=\ell)$ is a positive real sequence. In fact, this idea was proposed, first, by Fan & Gijbels (1996) in nonfunctional setup. Under this consideration, our motivation is based on the fact that the smooth kNN-LLM of the CCDF is obtained by estimating the operators *a*_*yx*_ and *b*_*yx*_ of the formula (1) as

$Min_{(a,b)\in {\mathrm {I\!R}} ^2}\sum_{i=1}^n\left(H(\ell_l^{-1}(y-Y_i))-a-bd(X_i,x)\right)^{\! 2}Ker\left(\frac{d(x,X_i)}{{ \mathrm {I\!H}}_k}\right),$

where ${\ell _l} = \min \{ \ell \in {\kern 1pt} \;{ \mathrm {I\!R}} ^ + ,{\kern 1pt} \;\text{satisfies}\;{\kern 1pt} {\sum\nolimits_{i = 1}1 _{(y - \ell ,y + \ell )}}({Y_i}) = l\}$. Then, we prove that the smooth kNN-LLM of the CCDF *F*(*y*|*x*) is explicited by

$\widehat{F} (y|x)={\sum_{{i,j=1}}^n \beta_{ij}H(\ell_l^{-1}(y-Y_i))\over{\sum_{{i,j=1}}^n \beta_{ij}}}.$

## Results and discussions

### On the impact of this contribution

It is well known that the CCDF has pivotal role in nonparametric statistics modeling. Indeed, the nonparametric estimation of this model is an imperative step for several nonparametric model including conditional density, the conditional quantile functions and the conditional hazard. In the prediction setting, the CCDF allows constructing various predictive regions or, more specifically, predictive intervals. We mention for instance, the shortest conditional modal interval (SCMI), the conditional percentile interval and the maximum conditional density region (MCDR) (see De Gooijer & Gannoun (2000) for their definitions). Of course, the diversity of the applicability of CCDF highlights the importance of this conditional model, which has the power of characterizing, completely, the conditional law of the considered random variables. As mentioned in the bibliographical discussion of the introduction section, the CCDF model has been widely studied in NPFDM. However, our present work’s novelty mainly estimates the CCDF model based on the combination of two fundamental approaches: the *k*NN and the LLM. This combination allows to construct an attractive estimator allowing to inherits the advantages of two methods. Indeed, it is well known that the LLM improves the bias property of the CKM while the weighting by the kNN-algorithm offers a sophisticated procedure for the smoothing parameter choose. It is selected locally with respect to the vicinity at the point of conditioning which is more adaptive to the data topological structure. Such adaptation is essential in nonparametric functional data analysis, where our estimators’ efficiency is connected to the data structure explored through the concentration property of the probability measure of the functional variable (see Ferraty & Vieu (2006)). Nevertheless, the establishment of the convergence rate of the kNN-LLM estimators is more complicated than the case considered by Laksaci, Rachdi & Rahmani (2013). In our case, the smoothing parameter is taken to be a random variable, while it is a scalar in the classical situation. Considering the dependent case which is more general and more realistic situation this difficulty becomes more complicated. In conclusion, the principal axes of this contribution are: (1) the conditional distribution function as a pivotal model for various nonparametric conditional models, (2) the estimation method as a new proceeder even in the nonfunctional case (as far as we know, there is no work in the CCDF estimation by combining the LLM to kNN) and (3) the functional time series case as a generalization for the independent case. To emphasize the usefulness of the present contribution in the prediction issue, we discuss in the following section how we can predict real future characteristic of a continuous-time process given its past.

### Functional time series prediction

Recall that the nonparametric prediction is considered to be the most important application of the functional nonparametric data analysis. In particular, functional time series examples can be composed based on a continuous-time process. Indeed, consider a random variable (*S*_*t*_) where *t* ∈ [0, *b*) having real-values in a continuous-time process. So, from *S*_*t*_ we compose *n* functional random variables (*X*_*i*_)_*i*__= 1,…,*n*_ obtained by

$\forall t\in [0,b),\qquad X_i(t)=S_{n^{-1}((i-1)b+t)}.$

Therefore, if our aim is to predict a future value *Y* = $S_{t_{0}}$, at fixed point *t*_0_ = *b* + *s* given (*S*_*t*_)_*t*__∈__[0,__*b*)_, we then define a sequence of the interest variable *Y*, i.e.,

$Y_i=S_{n^{-1}(i)b+s}, \qquad i=1,\ldots, n.$

Thereafter, we construct our predictor (conditional median, conditional quantile or the conditional mode) by using the observations (*X*_*i*_, *Y*_*i*_)_*i*__= 1,…,__*n*__−__1_. However, since the predictive region or, more specifically, the predictive interval is often more instructive than predicting a single-point, we focus on this kind of prediction. Formally, for all *ζ* ∈ (0, 1), the interval/region is defined as a set ${I_\zeta } \subset {\rm I}{\rm R}$ satisfies

${\mathrm {\rm I\!P}}\left(Y_n\in I_\zeta|X_n\right)=1-\zeta.$

As mentioned in the above section, one of the main feature of the CCDF is the possibility to construct several predictive regions *I*_*ζ*_. Of course, the efficiency of each prediction interval is assessed by the means of the length of the set *I*_*ζ*_ and the presence of the true value in *I*_*ζ*_. It is well documented that the width of the SCMI is the smallest compared to all predictive regions with the same coverage probability (see De Gooijer & Gannoun (2000)). The latter is introduced by Tong & Yao (1995) and obtained by

$[A_{1-\zeta}, B_{1-\zeta}]=\arg\min_{c,d}\left\{Leb[c,d]\,|\,\quadF(d|x)-F(c|x)\geq 1-\zeta\right\}.$

The *Leb*(·) refers to the Lebesgue measure. Using the CCFD estimators, we approximate the SCMI by

$[A_{1-\zeta}(X_n), B_{1-\zeta}(X_n)]=\arg\min_{c,d}\left\{Leb[c,d]|\,\quad\ddot{F}(d|{X_n})-\ddot{F}(c|{X_n})\geq 1-\zeta\right\}.$

where $\ddot F$ means $\widehat F$ or *$\tilde{F}$*. The easy implementation of this approximation is studied and discussed in the “REAL-DATA APPLICATION” section.

## Simulation study

In this simulation study, we propose to control the behaviour of the estimators w.r.t. the dependency degree of the data. More precisely, our aim is to compare, considering finite sample, the efficiency of the estimators $\widehat F(y|x)$ and $\tilde{F}$(*y*|*x*). To do this, we use the fact that *m*-dependent variables are *α*-mixing and we generate *n* functional variables as follows.

In the first, we draw *n* + *m* − 1 independent functional variables by

$S_j(t)=2\cos(tW_{1_j})+0.2W_{2_j} \hbox{ for } j=1, \ldots, n+m-1,$

where W1 and W2 are uniformly distributed on [0, π/4]. Next, we simulate the m-dependent functional variables defined by:

$X_i(t)=\sum_{j=i}^{i+m-1}S_j(t).$

**Figure 1:**
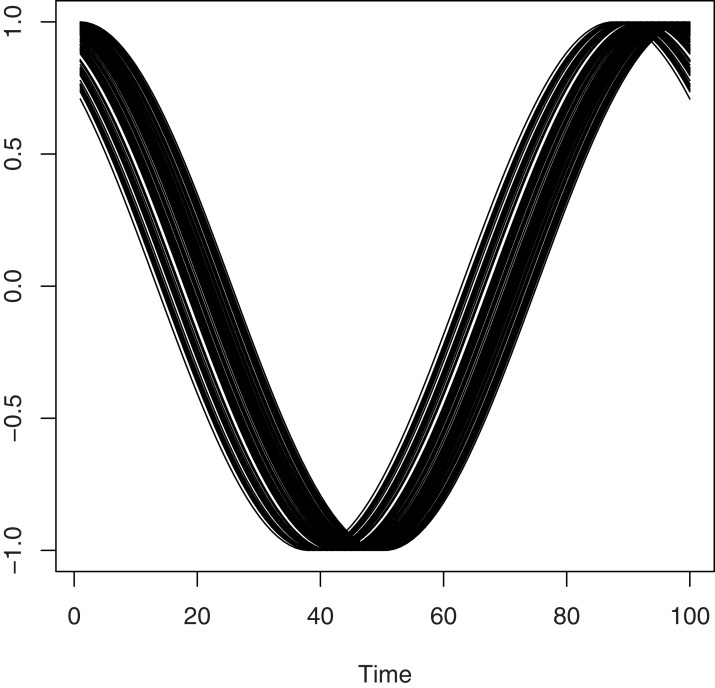
A sample of 100 curves.

Discretizing the curves *X*_*i*_’s on the same grid leads to the construction of 100 equispaced measurements in (0, 2*π*). In Fig. 1, we plotted the functional variables associated to the strong case where *m* = 4.

For the response variable we consider four regression models:

${\kern 1pt} \;Model\;M1{\kern 1pt} :{Y_i} = 5\int_0^{2\pi } \log ((4 - {X_i}(t{))^2} + 2)dt + {\varepsilon _i},$

${\kern 1pt} \;Model\;M2{\kern 1pt} :{Y_i} = \int_0^{2\pi } \exp ( - {X_i}(t))dt + 1.5\int_0^{2\pi } \exp ({X_i}(t))dt + {\varepsilon _i},$

$\eqalignb{ \;Model\;M3{\kern 1pt} :{Y_i} = \int_0^{2\pi } (5\log ((4 - {X_i}(t{))^2} + 2))dt + \int_0^{2\pi } \exp ( - {X_i}(t))dt\cr+ 1.5{\delta _i}\int_0^{2\pi } \exp ({X_i}(t))dt,$

$\eqalignb{ \;Model\;M4{\kern 1pt} :{Y_i} = \int_0^{2\pi } \exp ( - {X_i}(t))dt + 1.5\int_0^{2\pi } \exp ({X_i}(t))dt\cr+ 5{\delta _i}\int_0^{2\pi } \log ((4 - {X_i}(t{))^2} + 2)dt,$

where *ε*_*i*_ ∼ *N*(0,0.25) (resp. *δ*_*i*_ ∼ *Exp*(2)). Note that, based on this models and with given *X* = *x*, the CCDF of *Y* is explicitly determined according to the distributions of *ε*_*i*_ and *δ*_*i*_, which permit the determination of the theoretical CCDF, *F*(*y*|*x*).

Now, we specify quickly the different parameters involved in both estimators. Note that the parameters of the two estimators are the kernel *K*, the semi-metric *d*, the number *k* and/or *l* of neighbors. So, for this numerical study, we point out that we have taken a quadratic kernel supported within (0, 1) and used the *L*_2_ metric and the numbers of neighbors *k*, *l* are chosen using the following cross-validation criterion, defined as

$\sum_{ j=1}^n\left(F(Y_j|X_j)-\bar{F}^{-j}(Y_j|X_j)\right),$

where $\bar{F}^{-j}$ denotes the leave-one-out-curve estimate of the $\tilde{F}$ and $\widehat F$.

The performances of the two estimates is examined by comparing their average absolute errors:

${ AE}(\tilde{F})= \frac{1}{n}\sum_{i=1}^{n}\left| F(Y_i|X_i)-\tilde{F}(Y_i|X_i) \right| {\ and\  }{AE}(\tilde{F})=\frac{1}{n}\sum_{i=1}^{n}\left| F(Y_i|X_i)-\widehat{F}(Y_i|X_i) \right|.$

**Figure 2:**
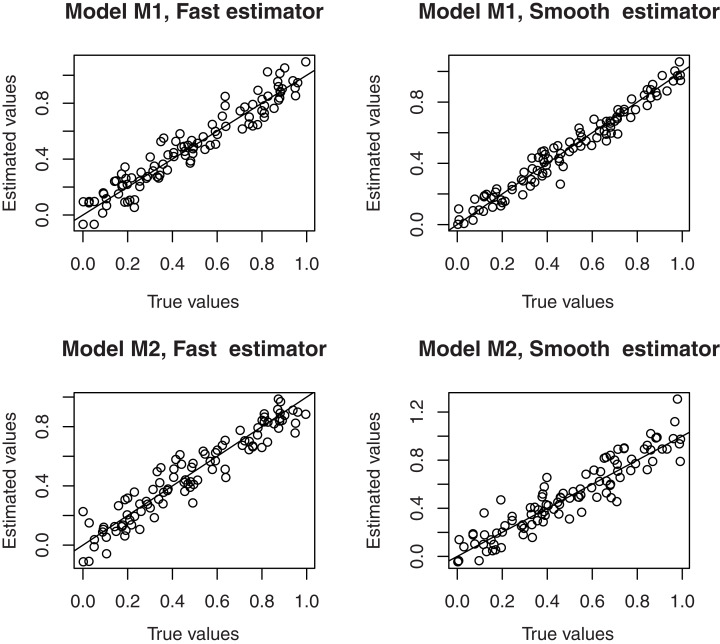
AE($\tilde{F}$) (dotted line) and AE($\widehat F$) (continuous line).

Thus, in order to control behaviors of both estimates w.r.t. the level of dependency, we plotted in Fig. 2, the curves of *AE*($\tilde{F}$) and *AE*($\widehat F$) w.r.t. the values of *m*.

It can be seen that, both errors increase substantially relatively to the values of *m*. Furthermore, it is clear that in the models M3 and M4 (heteroscedastic case), the estimate $\widehat F$ outperforms $\tilde{F}$, but in the models M1 and M2 (homoscedastic case), the estimate $\tilde{F}$ is significantly better than $\widehat F$.

## Real-data application

**Figure 3:**
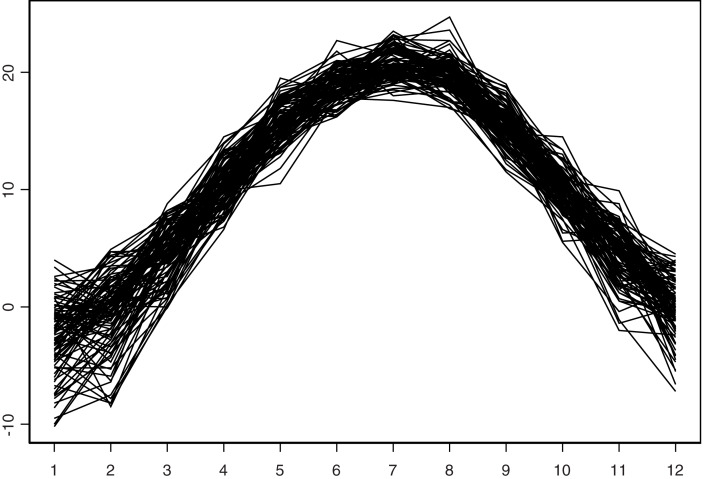
Mean temperature by year.

**Figure 4:**
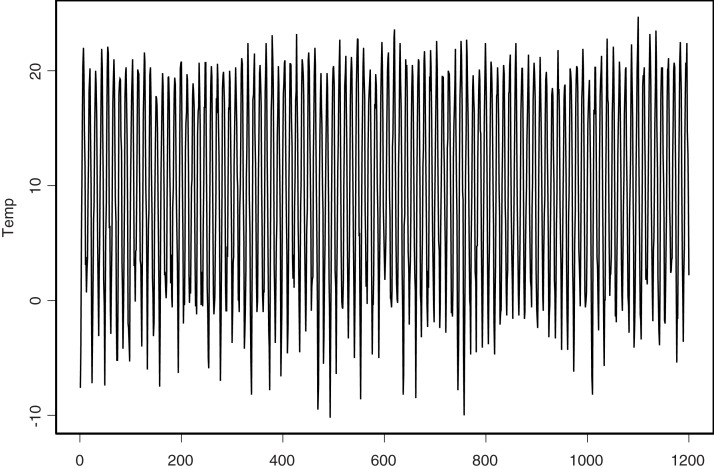
Monthly mean temperature.

In this section, we show the applicability of the proposed estimators to a real data example. To do that, we consider the problem of predicting the monthly average temperature one year ahead. For this purpose, we consider the same data set used by Laksaci, Lemdani & Said (2011) which are available at the website https://www.met.hu/en/eghajlat/magyarorszag_eghajlata/eghajlati_adatsorok/Debrecen/adatok/napi_adatok/index.php. This data were collected by Debrecen’s station, Hungary (northern latitude 47°35′44″ and eastern longitude 21°38′43″). They are monthly measurements (1,200 months = 100 years) from 1901 to 2000. The latter can be viewed as a continuous process denoted by *S*_*t*_. As noticed in the previous section, from *S*_*t*_, we construct *n* + 1 = 100 curves (*X*_*i*_(*t*)), *i* = 1,…, *n* + 1, where *X*_*i*_ denotes the average temperature curve observed during the (1 year) 12 months of the *i*^*th*^ year. The process (*S*_*t*_) and the curves (*X*_*i*_)_*i*_ are plotted in Figs. 3 and 4.

Of course, the proposed predictive interval’s efficiency is closely connected to the parameters’ choices in the estimator of the conditional distribution function. For the real data example, we compare estimators *F* and *F* in the SCMI estimation (with *ζ* = 0.1). For the computational study, we use the same kernel *K*, that is, the quadratic kernel on (0,1). The latter is adequate with this type of nonparametric approach. It is usually used in nonparametric functional statistics and incorporates the technical assumptions of the theoretical development of this kind of model. Concerning the choice of the metric *d*, we point out that it is closely related to the nature of the functional variable and its smoothing property. The Principal Component Analysis (PCA) metric is more suitable for this type of discontinuous functional regressors. For the choice of *k* (or *h*), we utilize the same cross-validation method used by De Gooijer & Gannoun (2000), which is based on the criterion

$CV=\frac{1}{n}\sum_{j=1}^{n}\sum_{i=1}^{n} (\hbox{1} _{\{Y_i\leq Y_j\}}-\widehat{F} ^{-j}(Y_i|x_j))^2 \ .$

This criterion is optimised over the same subsets of *k* (or *h*) proposed by Rachdi & Vieu (2007), that is, {5,10,20,…,45}.

**Figure 5:**
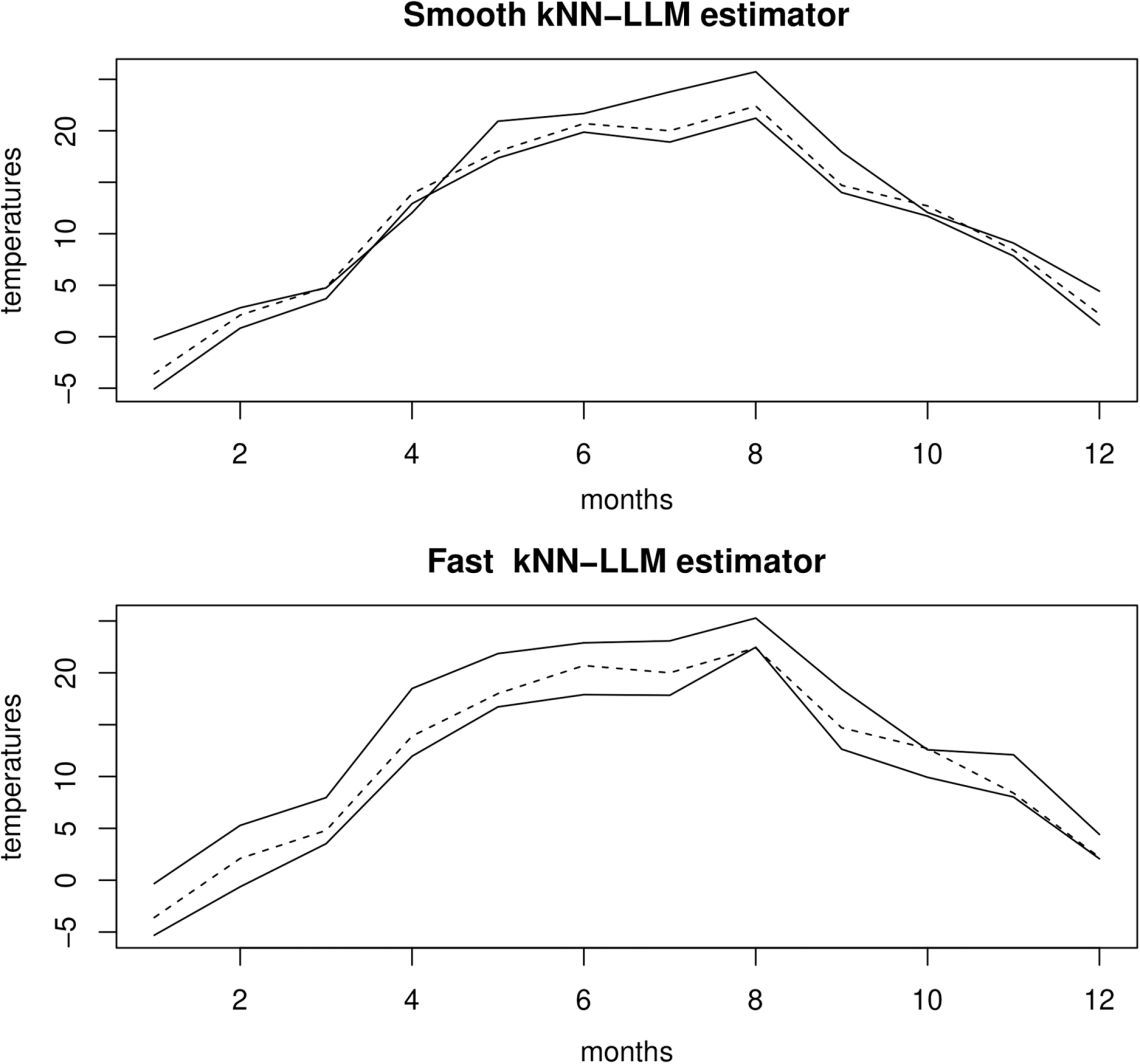
Results obtained by smooth kNN-LLM and fast kNN-LLM estimators.

To determine the SCMI predictive interval of the whole curve of the last year (*i* = 100) of this sample knowing the functional covariates *X*_99_, we use the first 98 curves as a training sample. Then, we predict the CCDF knowing *X*_99_ by $\widehat F$(·|*X*_99 *_) and $\tilde{F}$(·|*X*_99*_) where *X*_99*_ is the nearest curve to *X*_99_ in the training sample ${(Y_i^j,{X_i})_{i = 1 \ldots 99}}$ with $Y_i^j = {X_{i + 1}}(j)$, for each fixed month *j* = 1,…,12. Figure 5 displays the results. The dashed curve represents the observed data and the solid curves represent the estimated values for the two extremities of the SCMI predictive interval. We observe that the result of the $\tilde{F}$ is significantly better than the $\widehat F$ one. In the sense that it has an average mean length (*M.L* = 1.23) versus M.L = 1.97 for $\widehat F$. Of course, this gain is not surprising.

In the second illustration, we emphasize the importance of the kNN-LLM estimation of CCDF on the construction of the SCMI predictive interval by comparing the two kNN-LLM estimators, *f* and $\widehat F$, to their competitive estimators constructed by the kNN-Nadraya–Watson (kNN-NW) method. More precisely, we compare the estimators $\tilde{F}$ and $\widehat F$ to the smooth ($\tilde{F}$_1_(*y*|*x*)) and fast ($\widehat F$_1_(*y*|*x*)) kNN-NW estimators, where

$\widehat{F}_1(y|x)= \frac{\sum_{{i=1}}^n Ker\left({d(x,X_i)}{{ \mathrm {I\!H}}_k}\right)H(\ell_l^{-1}(y-Y_i))\over{\sum_{{i}}^n Ker\left(\frac{d(x,X_i)}{{ \mathrm {I\!H}}_k}\right)}}$

and

$\widehat{F}_1(y|x)= \frac{\sum_{{i=1}}^n Ker\left(\frac{d(x,X_i)}{{ \mathrm {I\!H}}_k}\right) \hbox{1} _{\{Y_1\leq y\}}\over{\sum_{{i=1}}^n Ker\left(\frac{d(x,X_i)}{{ \mathrm {I\!H}}_k}\right)}}.$

**Figure 6:**
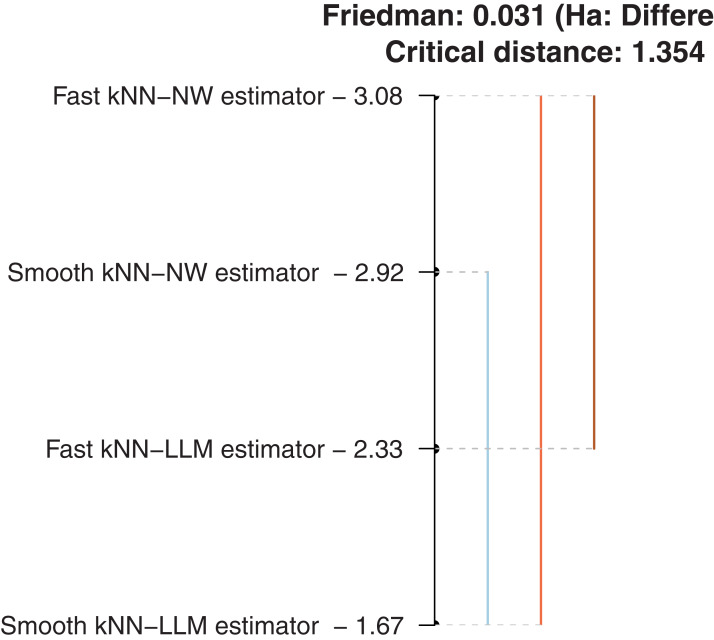
Comparison of the average means of length intervals.

Of course, in order to conduct a comprehensive comparison, we must treat the four models. For this reason, we have used the same kernel, the metric as well as the same selection method of the optimal number of neighbor *k*. Similar to the first illustration, this comparative analysis is performed over the 12 functional time series ${(Y_i^j,{X_i})_{i = 1 \ldots 100}}$ with $Y_i^j = {X_{i + 1}}(j)$, for each fixed month *j* = 1,…,12. For each fixed *j*, we split (randomly) the functional time series ${(Y_i^j,{X_i})_{i = 1 \ldots 100}}$ into two parts: the learning sample (70 observations) and the test sample (30 observations). Next, we compute the SCMI predictive intervals for the curves of the testing sample using the estimators $\tilde{F}$, $\widehat F$, $\tilde{F}$_1_ and $\widehat F$_1_. The efficiency of the four models is evaluated using the Nemenyi test plots for the average mean of the interval-length (ML). The comparison results are displayed in Fig. 6.

Once again, the conclusion is without surprise. It confirms the statement mentioned in the first illustration. More precisely, the local linear approach is more accurate than the Nadaraya-Watson method. This conclusion emphasizes the superiority of the local linear over the classical kernel method, which has been shown in the multivariate statistics through the bias term. On the other hand, it should be noted that all these four functional approaches have a stratification performance in this context of predictive issues.

## Conclusion

In this paper, we have studied the nonparametric estimation of the cumulative distribution function of the scalar response variable given a functional explanatory variable. Two new estimators are constructed by combining the local linear approach to the kNN smoothing methods. The first one is built by a fast algorithm based on the conditional cumulative distribution as classical regression of the indicator function. The second estimator is obtained by integration of the double kernel conditional density estimator. The latter gives a smooth estimator of the conditional cumulative distribution function. An empirical analysis is conducted to compare both estimators and their easy implementation in practice. Both artificial and real data carry out the finite sample performance of the two estimators. Undoubtedly, the present contribution highlights the conditional distribution function’s potential impact as a pivotal model in functional data analysis. It is involved in various conational nonparametric models, and its estimation is indispensable as preliminary steps to estimate numerous nonparametric functional models. For instance, we have focused on the prediction problem in this paper, and we have constructed two predictors (single prediction and region predictor). The artificial data analysis shows that both estimators have a satisfactory efficiency in different sinarios of regression data analysis, including homoscedasticity case, heteroscedasticity case, mixture models case, light-tailed or heavy-tailed conditional distribution cases. In conclusion, we can say that the functional data analysis through the conditional distribution has a significant impact in practice, and the proposed estimators of this contribution are fast, very easy to implement, and have a good performance in the prediction issues. Finally, let us note that the present contribution opens several prospects for future research. For example, it will be very interesting to compare the efficiency of our approach to other functional models such as the robust regression and the relative regression. Such models allow to reduce the sensitivity of the kNN approach to the noisy data, missing values, and the presence of outliers.

## Supplemental Information

10.7717/peerj.11719/supp-1 Supplemental Information 1 R code.

## Additional Information and Declarations

### Data Availability

The following information was supplied regarding data availability: The data used in our paper is available at the Hungarian Meteorological Service: https://www.met.hu/en/eghajlat/magyarorszag_eghajlata/eghajlati_adatsorok/Debrecen/adatok/napi_adatok/index.php The code is available as a Supplemental File.
